# Supplementary material for: Transitions in dynamical regime and neural mode during perceptual decisions
Source: Nature. 2025 Sep 17;646(8087):1156–66. doi: 10.1038/s41586-025-09528-4 (PMC12571898; doi:10.1038/s41586-025-09528-4)
Supplement: Supplementary file 2 — Reporting Summary [file 41586_2025_9528_MOESM2_ESM.pdf]

nature portfolio

Corresponding author(s): Thomas Luo (zhihaol@princeton.edu), Timothy Kim (timkimd@uw.edu), Carlos Brody (brody@princeton.edu)

Last updated by author(s): 07/1/2025

Reporting Summary

Nature Portfolio wishes to improve the reproducibility of the work that we publish. This form provides structure for consistency and transparency in reporting. For further information on Nature Portfolio policies, see our Editorial Policies and the Editorial Policy Checklist.

Statistics

For all statistical analyses, confirm that the following items are present in the figure legend, table legend, main text, or Methods section.

n/a	Confirmed	
☐	☒	The exact sample size (*n*) for each experimental group/condition, given as a discrete number and unit of measurement
☐	☒	A statement on whether measurements were taken from distinct samples or whether the same sample was measured repeatedly
☐	☒	The statistical test(s) used AND whether they are one- or two-sided<br>*Only common tests should be described solely by name; describe more complex techniques in the Methods section.*
☐	☒	A description of all covariates tested
☐	☒	A description of any assumptions or corrections, such as tests of normality and adjustment for multiple comparisons
☐	☒	A full description of the statistical parameters including central tendency (e.g. means) or other basic estimates (e.g. regression coefficient) AND variation (e.g. standard deviation) or associated estimates of uncertainty (e.g. confidence intervals)
☐	☒	For null hypothesis testing, the test statistic (e.g. *F*, *t*, *r*) with confidence intervals, effect sizes, degrees of freedom and *P* value noted<br>*Give P values as exact values whenever suitable.*
☐	☒	For Bayesian analysis, information on the choice of priors and Markov chain Monte Carlo settings
☒	☐	For hierarchical and complex designs, identification of the appropriate level for tests and full reporting of outcomes
☒	☐	Estimates of effect sizes (e.g. Cohen's *d*, Pearson's *r*), indicating how they were calculated

Our web collection on statistics for biologists contains articles on many of the points above.

Software and code

Policy information about availability of computer code

Data collection	https://github.com/Brody-Lab/decision_dynamics_commitment/tree/main/code/data_collection
Data analysis	Data were analyzed using code in the following repositories:<br>1) https://github.com/Brody-Lab/decision_dynamics_commitment<br>2) https://github.com/Brody-Lab/findr<br>3) https://github.com/Brody-Lab/FHMDDM<br>4) https://github.com/Brody-Lab/tzl_spGLM

For manuscripts utilizing custom algorithms or software that are central to the research but not yet described in published literature, software must be made available to editors and reviewers. We strongly encourage code deposition in a community repository (e.g. GitHub). See the Nature Portfolio guidelines for submitting code & software for further information.

Data

Policy information about availability of data

All manuscripts must include a data availability statement. This statement should provide the following information, where applicable:

- Accession codes, unique identifiers, or web links for publicly available datasets
- A description of any restrictions on data availability
- For clinical datasets or third party data, please ensure that the statement adheres to our policy

The experimental data that support the findings of this study are available in Dryad with the identifier https://doi.org/10.5061/dryad.sj3tx96d

Research involving human participants, their data, or biological material

Policy information about studies with human participants or human data. See also policy information about sex, gender (identity/presentation), and sexual orientation and race, ethnicity and racism.

Reporting on sex and gender	N/A
Reporting on race, ethnicity, or other socially relevant groupings	N/A
Population characteristics	N/A
Recruitment	N/A
Ethics oversight	N/A

Note that full information on the approval of the study protocol must also be provided in the manuscript.

Field-specific reporting

Please select the one below that is the best fit for your research. If you are not sure, read the appropriate sections before making your selection.

☒ Life sciences ☐ Behavioural & social sciences ☐ Ecological, evolutionary & environmental sciences

For a reference copy of the document with all sections, see nature.com/documents/nr-reporting-summary-flat.pdf

Life sciences study design

All studies must disclose on these points even when the disclosure is negative.

Sample size	Sample sizes were chosen based on conventions in the field of systems neuroscience, where comparable electrophysiological studies in rats typically include 3-12 rats (12 in this study) and 48-200 sessions (here 115), as in Hanks et al., 2015 (Nature); Yartsev et al., 2018 (eLife); Pagan et al., 2025 (Nature). No formal power analysis was conducted, as the goal was to discover robust effects in a biologically realistic context rather than test a specific effect size. The number of animals and sessions reflects a balance between statistical power and the feasibility of time-intensive recordings and behavioral training.
Data exclusions	Electrophysiological data in time periods beyond the relevant epoch of the behavior being studied were excluded
Replication	We repeated the electrophysiological recording in 115 separate sessions, each involving a single animal, with a total of 12 animals (some contributing to multiple sessions). Each session served as an independent replicate.
Randomization	All animal subjects were part of a single experimental group.
Blinding	Blinding was not relevant because all animal subjects form a single experimental group.

Reporting for specific materials, systems and methods

We require information from authors about some types of materials, experimental systems and methods used in many studies. Here, indicate whether each material, system or method listed is relevant to your study. If you are not sure if a list item applies to your research, read the appropriate section before selecting a response.

Materials & experimental systems

n/a	Involved in the study
☒	☐ Antibodies
☒	☐ Eukaryotic cell lines
☒	☐ Palaeontology and archaeology
☐	☒ Animals and other organisms
☒	☐ Clinical data
☒	☐ Dual use research of concern
☒	☐ Plants

Methods

n/a	Involved in the study
☒	☐ ChIP-seq
☒	☐ Flow cytometry
☒	☐ MRI-based neuroimaging

Animals and other research organisms

Policy information about studies involving animals; ARRIVE guidelines recommended for reporting animal research, and Sex and Gender in Research

Laboratory animals	Rattus norvegicus, Long-Evans, 6 months to 24 months old
Wild animals	No wild animal was used in the study
Reporting on sex	Only male rats were used for this study because their larger body is necessary for the implants used to collect large-scale electrophysiological data. Similar previous studies using rats in systems neuroscience focused on using only male rats.
Field-collected samples	No field-collected samples were used in the study
Ethics oversight	The animal procedures described in this study were approved by the Princeton University Institutional Animal Care and Use Committee and were carried out in accordance with National Institutes of Health standards.

Note that full information on the approval of the study protocol must also be provided in the manuscript.

Plants

Seed stocks	*Report on the source of all seed stocks or other plant material used. If applicable, state the seed stock centre and catalogue number. If plant specimens were collected from the field, describe the collection location, date and sampling procedures.*
Novel plant genotypes	*Describe the methods by which all novel plant genotypes were produced. This includes those generated by transgenic approaches, gene editing, chemical/radiation-based mutagenesis and hybridization. For transgenic lines, describe the transformation method, the number of independent lines analyzed and the generation upon which experiments were performed. For gene-edited lines, describe the editor used, the endogenous sequence targeted for editing, the targeting guide RNA sequence (if applicable) and how the editor was applied.*
Authentication	*Describe any authentication procedures for each seed stock used or novel genotype generated. Describe any experiments used to assess the effect of a mutation and, where applicable, how potential secondary effects (e.g. second site T-DNA insertions, mosiacism, off-target gene editing) were examined.*
